# Post-diagnosis weight trajectories and mortality among women with breast cancer

**DOI:** 10.1038/s41523-023-00603-5

**Published:** 2023-12-02

**Authors:** Leah S. Puklin, Fangyong Li, Brenda Cartmel, Julian Zhao, Tara Sanft, Alexa Lisevick, Eric P. Winer, Maryam Lustberg, Donna Spiegelman, Mona Sharifi, Melinda L. Irwin, Leah M. Ferrucci

**Affiliations:** 1 Yale School of Public Health, Yale University, New Haven, CT 06510 USA; 2 Yale Cancer Center, New Haven, CT 06510 USA; 3 Yale University School of Medicine, 333 Cedar St., New Haven, CT 06520 USA; 4 Medical College of Wisconsin, Milwaukee, WI 53226 USA

**Keywords:** Breast cancer, Breast cancer, Epidemiology, Cancer epidemiology

## Abstract

Weight gain after breast cancer diagnosis is associated with adverse health outcomes. Yet, few studies have characterized post-diagnosis weight change in the modern treatment era or populations most at risk for weight changes. Among women diagnosed with stages I–III breast cancer in the Smilow Care Network (2013–2019; *N* = 5441), we abstracted demographic and clinical characteristics from electronic health records and survival data from tumor registries. We assessed if baseline characteristics modified weight trajectories with nonlinear multilevel mixed-effect models. We evaluated body mass index (BMI) at diagnosis and weight change 1-year post-diagnosis in relation to all-cause and breast cancer-specific mortality with Cox proportional hazard models. Women had 34.4 ± 25.5 weight measurements over 3.2 ± 1.8 years of follow-up. Weight gain was associated with ER/PR−, HER2+ tumors, BMI ≤ 18.5 kg/m^2^, and age ≤ 45 years (+4.90 kg (standard error [SE] = 0.59), +3.24 kg (SE = 0.34), and +1.75 kg (SE = 0.10), respectively). Weight loss was associated with BMI ≥ 35 kg/m^2^ and age ≥ 70 years (−4.50 kg (SE = 0.08) and −4.34 kg (SE = 0.08), respectively). Large weight loss (≥10%), moderate weight loss (5–10%), and moderate weight gain (5–10%) 1-year after diagnosis were associated with higher all-cause mortality (hazard ratio [HR] = 2.93, 95% confidence interval [CI] = 2.28–3.75, HR = 1.32, 95% CI = 1.02–1.70 and HR = 1.39, 95% CI = 1.04–1.85, respectively). BMI ≥ 35 kg/m^2^ or BMI ≤ 18.5 kg/m^2^ at diagnosis were also associated with higher all-cause mortality. Weight change after a breast cancer diagnosis differed by demographic and clinical characteristics highlighting subgroups at-risk for weight change during a 5-year period post-diagnosis. Monitoring and interventions for weight management early in clinical care are important.

## Introduction

Change in body weight after a breast cancer diagnosis has been of interest for several decades, with one of the first reports from 1978 describing substantial weight gain during adjuvant chemotherapy^1^. Subsequent studies found that 50–96% of women receiving adjuvant chemotherapy for breast cancer gained an average of 2.5–6.2 kg during treatment^1–6^. Longer treatment duration and chemotherapeutic regimens as well as treatment-related factors such as increased fatigue and taste-change, may explain some of this weight gain. In addition, for all women with breast cancer, even those not receiving chemotherapy, behavioral changes, including reduced physical activity levels and alterations in diet, may be relevant to weight gain after diagnosis^7–9^.

Obesity at diagnosis may negatively impact prognosis, yet evidence on weight change after breast cancer diagnosis and mortality is limited^10^. Excess adiposity at diagnosis has been shown to be associated with a higher risk of recurrence and mortality^11–13^. Data on post-diagnosis weight gain and survival have been less consistent, though evidence suggests large weight gain (≥10%) is associated with poorer survival^14,15^. Large weight loss after breast cancer diagnosis among patients diagnosed with stages I–III disease was associated with a 41% higher risk of overall mortality compared with weight stability over 2 years after diagnosis^16^. It is suggested that weight loss due to muscle loss (defined as sarcopenia) may, in part, explain this increased risk of mortality^17^.

Since the detection, diagnosis, and treatment of breast cancer have changed substantially over time, earlier studies of weight patterns after breast cancer may not be relevant to modern populations. As primary chemotherapeutic agents changed and treatment lengths shortened, observational studies started to note less pronounced weight gain^15,18–20^. Advancements in screening have also led to earlier detection and diagnosis of breast cancer resulting in fewer women being diagnosed with late-stage disease^21^. Additionally, tests to guide the use of chemotherapy have emerged, reducing the percentage of women receiving chemotherapy^22^.

Variations in analytical approaches and study populations have also made comparisons across studies challenging. Historically, studies of breast cancer patients assessed weight change as the difference between two time points which limited the detection of nonlinear weight patterns^23–27^. The timing of post-diagnosis weight measurement also varies across studies^20,28^. Several studies have used repeated weight measurements but were limited by smaller sample sizes^15,28,29^. Lastly, many studies relied on self-reported rather than objective measures of weight^16,25,30,31^ and most analyses were in existing prospective cohorts^15,20,31,32^ as opposed to population-based samples.

Given the adverse health outcomes associated with weight changes after breast cancer, updating evidence on post-diagnosis weight patterns with real-world data is critical. These findings can help inform clinical practice by generating awareness around monitoring weight change in the short and long term, identifying populations most at risk for post-diagnosis weight change, and supporting the design and implementation of tailored weight management lifestyle interventions. Here we provide a contemporary evaluation of weight trajectories over a 5-year period post breast cancer diagnosis in a large sample of patients from a major healthcare system in Connecticut using robust longitudinal methods and clinically measured repeated weight data from electronic health records (EHRs).

## Results

### Patient characteristics

**Fig. 1:**
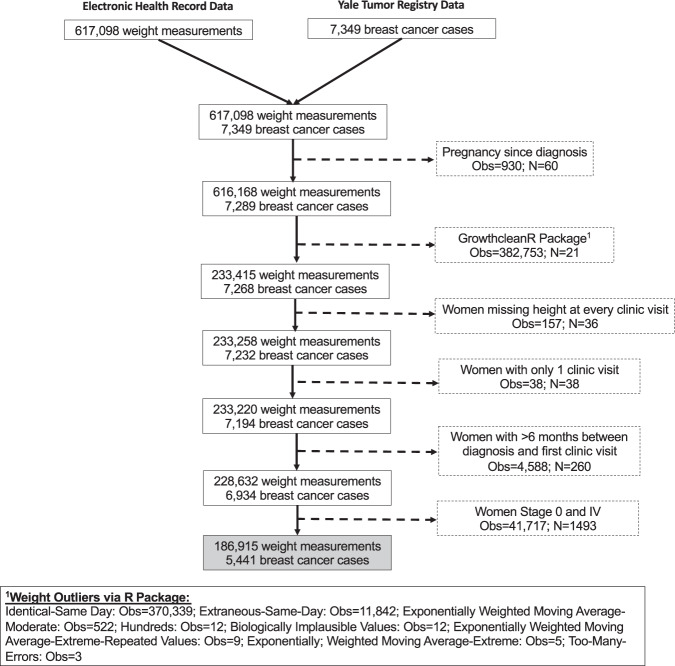
Flow diagram. Flow diagram describing study sample from Yale Tumor Registry-Smilow Cancer Hospital linked dataset and exclusions.

Among the 5441 women with stages I–III breast cancer in our dataset, the average duration of follow-up was 3.2 ± 1.8 years (±standard deviation [SD]) (range = 0.01–7.2 years) with a mean of 34.4 ± 25.5 weight measurements (Fig. 1 and Table 1). At diagnosis, the mean age was 61.0 ± 13.2 years and mean body mass index (BMI) was 29.3 ± 6.9 kg/m^2^. Most patients were Non-Hispanic white (80.3%), had stage I disease (63.7%), had ER/PR+, HER2− tumors (68.5%), did not receive chemotherapy (60.6%), received radiation therapy (62.9%), and had breast-conserving surgery (63.1%).

**Table 1:** Characteristics of women with breast cancer diagnosed from 2013 to 2019.

Characteristics	Overall^a,b^ (*N* = 5441)
Length of follow-up, years	3.2 ± 1.8 (0.01–7.2)
Number of weights	34.4 ± 25.5 (2–310)
Time from Dx to first weight measure, months	0.8 ± 0.8 (0–6.0)
Age at diagnosis, years	61.0 ± 13.2 (22–100)
Age at diagnosis, *n*	
≤45	580 (10.7)
45–55	1354 (24.9)
55–70	2169 (39.9)
≥70	1338 (24.6)
BMI at diagnosis, kg/m^2^	29.3 ± 6.9 (14.2–74.3)
BMI group at diagnosis	
Underweight (≤18.5)	67 (1.2)
Normal (18.5–25)	1565 (28.9)
Overweight (25–30)	1672 (30.7)
Class I obesity (30–35)	1127 (20.7)
Class II obesity (≥35)	1010 (18.6)
Race and ethnicity	
Non-Hispanic white	4367 (80.3)
Non-Hispanic Black	488 (9.0)
Hispanic	359 (6.6)
Other/unknown	227 (4.2)
Clinical stage at diagnosis	
Stage I	3465 (63.7)
Stage II	1325 (24.4)
Stage III	323 (5.9)
Unknown	328 (6.0)
Tumor subtype^c^	
ER/PR+, HER2−	3725 (68.5)
ER/PR+, HER2+	398 (7.3)
ER/PR−, HER2+	188 (3.5)
TNBC	524 (9.6)
Unknown	606 (11.1)
Chemotherapy	
No	3297 (60.6)
Yes	2144 (39.4)
Radiation	
No	2019 (37.1)
Yes	3422 (62.9)
Type of surgery	
No surgery	327 (6.0)
Breast-conserving surgery	3435 (63.1)
Mastectomy	1675 (30.8)
Endocrine therapy^d^	
No	1131 (20.8)
Yes	4310 (79.2)
Tamoxifen^e^	
No	4502 (82.7)
Yes	939 (17.3)
Aromatase inhibitor^e^	
No	1679 (30.9)
Yes	3762 (69.1)

*Dx* diagnosis, *BMI* body mass index, *TNBC* triple-negative breast cancer.

^a^Mean ± standard deviation, (range) for continuous variables, *n* (%) for categorical variables.

^b^Numbers may not sum to total due to missing data.

^c^Defined as ER/PR+, HER2− (ER+, PR+, HER2− or ER−, PR+, HER2− or ER+, PR−, HER2−), ER/PR+, HER2+ (ER+, PR+, HER2+ or ER+, PR−, HER2+ or ER−, PR+, HER2+), ER/PR−, HER2+ (ER−, PR−, HER2+), TNBC (ER−, PR−, HER2−).

^d^Defined as prescription of anastrozole, exemestane, tamoxifen citrate and letrozole any time in the follow-up period.

^e^Individuals could have been prescribed both an aromatase inhibitor and tamoxifen during the follow-up period.

### Weight trajectories by demographic and clinical characteristics

On average, women experienced modest weight change over 5 years post breast cancer diagnosis, (−0.80 kg (standard error [SE] = 0.04)) yet, post-diagnosis weight change differed significantly across several characteristics (Table 2).

**Table 2:** Weight at breast cancer diagnosis and weight change 1 year and 5 years post-diagnosis.

	Weight at dx, mean kg (se)^a,b^	*p*-value	Weight change in kg, dx to 1 year^b^	*p*-value	Weight change in kg, dx to 5 years^b^	*p*-value
Age at diagnosis, years						
≤45	71.9 (0.9)	Ref.	+1.00 (0.05)	Ref.	+1.75 (0.10)	Ref.
45–55	75.9 (0.7)	<0.001	+0.25 (0.03)	<0.001	+0.83 (0.07)	<0.001
55–70	77.1 (0.7)	<0.001	−0.09 (0.02)	<0.001	−0.93 (0.05)	<0.001
≥70	73.6 (0.7)	0.06	−0.91 (0.04)	<0.001	−4.34 (0.08)	<0.001
BMI group at diagnosis, kg/m^2^						
≤18.5	44.7 (1.2)	<0.001	+0.61 (0.15)	0.95	+3.24 (0.34)	<0.001
18.5–25	57.1 (0.4)	Ref.	+0.61 (0.03)	Ref.	+1.95 (0.07)	Ref.
25–30	69.4 (0.4)	<0.001	+0.28 (0.03)	<0.001	−0.27 (0.06)	<0.001
30–35	82.1 (0.4)	<0.001	−0.32 (0.03)	<0.001	−2.00 (0.08)	<0.001
≥35	100.9 (0.4)	<0.001	−1.01 (0.04)	<0.001	−4.50 (0.08)	<0.001
Race and ethnicity						
Non-Hispanic white	74.2 (0.5)	Ref.	+0.09 (0.02)	Ref.	−0.65 (0.04)	Ref.
Non-Hispanic Black	84.2 (0.9)	<0.001	−0.58 (0.05)	<0.001	−1.95 (0.11)	<0.001
Hispanic	74.3 (1.0)	0.92	−0.10 (0.06)	0.002	−0.64 (0.13)	0.93
Other/unknown	68.0 (1.2)	<0.001	−0.44 (0.09)	<0.001	−1.02 (0.18)	0.05
Clinical stage at diagnosis						
Stage I	75.0 (0.6)	Ref.	−0.03 (0.02)	Ref.	−0.84 (0.05)	Ref.
Stage II	75.0 (0.7)	0.95	+0.02 (0.03)	0.25	−0.65 (0.06)	0.02
Stage III	75.6 (1.1)	0.56	−0.14 (0.06)	0.06	−0.03 (0.12)	<0.001
Unknown	74.4 (1.1)	0.58	+0.25 (0.09)	0.003	−4.10 (0.21)	<0.001
Tumor subtype						
ER/PR+, HER2−	75.0 (0.7)	Ref.	−0.01 (0.02)	Ref.	−0.90 (0.04)	Ref.
ER/PR+, HER2+	72.4 (1.1)	0.01	+0.55 (0.05)	<0.001	+0.85 (0.11)	<0.001
ER/PR−, HER2+	75.1 (1.4)	0.93	+0.75 (0.07)	<0.001	+4.90 (0.59)	<0.001
TNBC	76.4 (1.0)	0.24	−0.60 (0.05)	<0.001	−1.63 (0.11)	<0.001
Unknown	75.6 (0.8)	0.49	−0.45 (0.07)	<0.001	−1.96 (0.15)	<0.001
Chemotherapy						
No	75.0 (0.7)	Ref.	−0.17 (0.02)	Ref.	−1.90 (0.05)	Ref.
Yes	75.0 (0.6)	0.97	+0.11 (0.02)	<0.001	+0.20 (0.05)	<0.001
Radiation						
No	73.5 (0.6)	Ref.	−0.04 (0.03)	Ref.	−0.91 (0.06)	Ref.
Yes	76.4 (0.6)	<0.001	0.00 (0.02)	0.30	−0.76 (0.04)	0.04

*Dx* diagnosis, *BMI* body mass index, *TNBC* Triple-negative breast cancer.

^a^Weight at dx is based on having a first weight within 6 months of dx.

^b^Adjusted for Age at diagnosis (continuous), Race and ethnicity, Tumor subtype (ER/PR+, HER2−; ER/PR+, HER2+; ER/PR−, HER2+, TNBC), Clinical stage, Chemotherapy (yes/no), Radiation (yes/no), Endocrine therapy (yes/no).

For age, over the first year after diagnosis, women ≥70 years lost more weight compared with women ≤45 years (−0.91 kg versus +1.00 kg, *p* < 0.001) (Table 2). Over 5 years, women ≤45 years gained +1.75 kg while women ≥70 years lost −4.34 kg (*p* < 0.001).

Post-diagnosis weight change also differed by BMI at diagnosis (Table 2). Over the first year after diagnosis, women with a BMI between 30 and 35 kg/m^2^ and a BMI ≥ 35 kg/m^2^ had significantly different weight changes compared with women with a BMI between 18.5 and 25 kg/m^2^ (−0.32 kg versus +0.61 kg, *p* < 0.001 and −1.01 kg versus +0.61 kg, *p* < 0.001, respectively). These trends continued for 5 years post-diagnosis. From diagnosis to 5 years post-diagnosis, women with a BMI ≤ 18.5 kg/m^2^ gained significantly more weight compared with women with a BMI between 18.5 and 25 kg/m^2^ (+3.24 kg versus +1.95, *p* < 0.001).

From diagnosis to 1-year post-diagnosis, Non-Hispanic Black and Hispanic women lost weight while Non-Hispanic white women remained weight stable (−0.58 kg versus +0.09 kg, *p* < 0.001 and −0.10 kg versus +0.09 kg, *p* = 0.002, respectively) (Table 2). By 5 years post-diagnosis, Non-Hispanic Black women still had a significant difference in weight loss compared with Non-Hispanic white women (−1.95 kg versus −0.65 kg, *p* < 0.001), but the difference was not statistically significant for Hispanic women (*p* = 0.93).

In the first year after diagnosis, women with ER/PR−, HER2+ tumors gained more weight than women with ER/PR+, HER2− tumors (+0.75 kg versus −0.01 kg, *p* < 0.001) and this trend extended over 5 years (+4.90 kg versus −0.90 kg, *p* < 0.001). Comparatively, women with triple-negative breast cancer lost more weight compared to women with ER/PR+, HER2− tumors at 1 year (−0.60 kg versus −0.01 kg, *p* < 0.001) and 5 years post-diagnosis (−1.63 kg vs. −0.90 kg, *p* < 0.001).

Women who did not receive chemotherapy lost −1.90 kg over 5 years, while women who received chemotherapy gained +0.20 kg (*p* < 0.001). Women who did not receive radiation therapy lost −0.91 kg over 5 years, compared with −0.76 kg among women who did receive radiation therapy (*p* = 0.04).

Weight change patterns in the sensitivity analysis including women with stage 0 and stage IV breast cancer were similar (Supplementary Table 1).

### One-year post-diagnosis weight change, BMI and mortality

Among the 4880 women with stages I–III breast cancer and a recorded weight measurement at 1-year post-diagnosis, 3079 (63.1%) remained weight stable, 355 (7.3%) lost ≥10% body weight, 631 (12.9%) lost 5–10% body weight, 590 (12.1%) gained 5–10% body weight and 225 (4.5%) gained ≥10% (Supplementary Table 2).

Compared with women who remained weight stable, large weight loss (≥10%) was associated with a higher risk of all-cause and breast cancer-specific mortality (HR = 2.93, 95% CI = 2.28–3.75, HR = 2.21, 95% CI = 1.42–3.43, respectively) (Table 3). Moderate weight loss (5–10%) was also positively associated with all-cause mortality (HR = 1.32, 95% CI = 1.02–1.70). Moderate weight gain (5–10%) 1-year after diagnosis was associated with a higher risk of all-cause and breast cancer-specific mortality (HR = 1.39, 95% CI = 1.04–1.85, HR = 1.80, 95% CI = 1.15–2.82, respectively). Results including women with stage 0 and IV disease and partitioned by median follow-up time are presented in Supplementary Tables 3 and 4.

**Table 3:** Cox proportional hazard model for weight change from diagnosis to 1 year and all-cause mortality, *N* = 4880.

	Large loss (≥10%)	Moderate loss (5–10%)	Stable (within 5%)	Moderate gain (5–10%)	Large gain (≥10%)
Breast cancer-specific	Events/*N*	Events/*N*	Events/*N*	Events/*N*	Events/*N*
167/4880	31/355	24/631	78/3079	26/590	8/225
Unadjusted	3.98 (2.63–6.04)	1.56 (0.99–2.46)	Ref.	1.73 (1.11–2.70)	1.40 (0.68–2.90)
Adjusted^a^	2.21 (1.42–3.43)	1.09 (0.68–1.74)	Ref.	1.80 (1.15–2.82)	1.15 (0.55–2.41)
All-cause mortality	Events/*N*	Events/*N*	Events/*N*	Events/*N*	Events/*N*
525/4880	95/355	79/631	273/3079	58/590	20/225
Unadjusted	3.64 (2.88–4.59)	1.50 (1.17–1.93)	Ref.	1.10 (0.83–1.46)	1.00 (0.63–1.57)
Adjusted^a^	2.93 (2.28–3.75)	1.32 (1.02–1.70)	Ref.	1.39 (1.04–1.85)	1.37 (0.87–2.18)

^a^Adjusted for Age at diagnosis (continuous), BMI at diagnosis (continuous), Race and ethnicity, Tumor subtype (ER/PR+, HER2−; ER/PR+, HER2+; ER/PR−, HER2+, TNBC), Clinical stage, Chemotherapy (yes/no), and Radiation (yes/no).

**Fig. 2:**
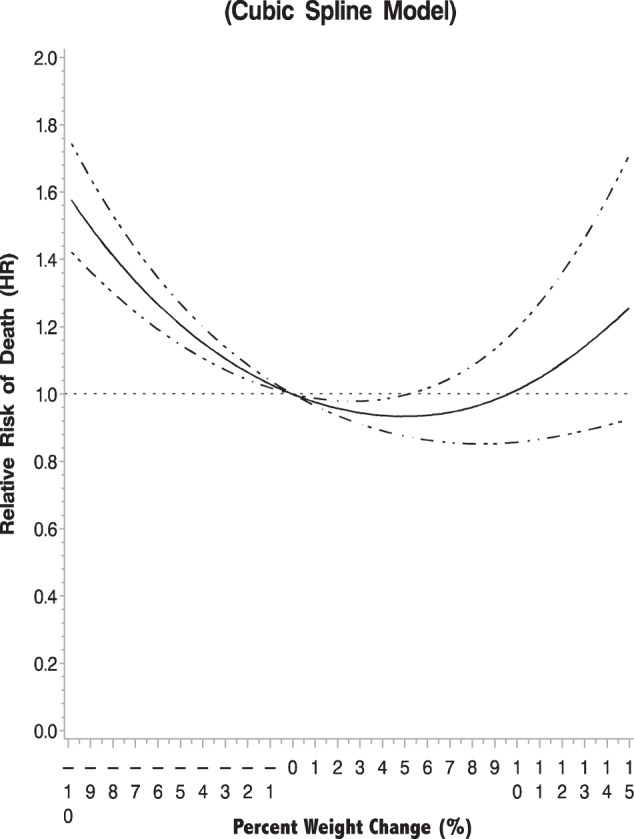
One-year post-diagnosis weight change and all-cause mortality using cubic spline model (*N* = 4880; deaths = 525). Model adjusted for age at diagnosis (continuous), race and ethnicity, clinical stage, subtype (ER/PR+, HER2−; ER/PR+, HER2+; ER/PR−, HER2+, TNBC), receipt of chemotherapy (yes/no), receipt of radiation (yes/no), and BMI group at diagnosis (continuous). Error bars represent 95% confidence interval.

There was a nonlinear relationship between percent weight change (continuous measure) and all-cause mortality in the cubic spline analysis (*p* < 0.001) (Fig. 2). Weight loss and the greatest weight gain were adversely associated with all-cause mortality.

Compared to women with a BMI between 18.5–25 kg/m^2^ at diagnosis, women with a BMI ≤ 18.5 kg/m^2^ had a higher risk of all-cause mortality (HR = 2.34, 95% CI = 1.27–4.34) (Table 4). Women with a BMI ≥ 35 kg/m^2^ at diagnosis also had an elevated risk of all-cause mortality (HR = 1.28, 95% CI = 1.00–1.62) compared with women with a BMI between 18.5 and 25 kg/m^2^. In the sensitivity analyses which included women with stage 0 and IV disease and women who died in the first year after diagnosis, a BMI ≤ 18.5 kg/m^2^ at diagnosis was also associated with a higher risk of breast cancer-specific mortality (Supplementary Tables 5 and 6).

**Table 4:** Association between BMI at diagnosis and breast cancer-specific and all-cause mortality via a Cox proportional hazards model, *N* = 5380.

	All-cause mortality (*N* = 571/5380)	Adjusted^a^ HR (95% CI)	*p*-value	Breast cancer-specific mortality (*N* = 185/5380)	Adjusted^a^ HR (95% CI)	*p*-value
BMI at diagnosis, kg/m^2^						
≤18.5 vs. 18.5–25	11/66	2.34 (1.27–4.34)	0.01	3/66	1.77 (0.55–5.71)	0.34
25–30 vs. 18.5–25	153/1652	0.86 (0.69–1.08)	0.20	55/1652	1.00 (0.68–1.49)	0.99
30–35 vs. 18.5–25	128/1119	0.95 (0.75–1.21)	0.67	40/1119	0.89 (0.58–1.37)	0.61
≥35 vs. 18.5–25	130/996	1.28 (1.00–1.62)	0.05	41/996	1.13 (0.74–1.74)	0.57
Continuous, per 5 kg/m^2^		1.05 (0.98–1.11)	0.15		0.97 (0.87–1.09)	0.62

^a^Adjusted for Age at diagnosis (continuous), Race and ethnicity, Tumor subtype (ER/PR+, HER2−; ER/PR+, HER2+; ER/PR−, HER2+, TNBC), Clinical stage, Chemotherapy (yes/no), and Radiation (yes/no).

## Discussion

In a large, population-based sample of 5441 women diagnosed with stages I–III breast cancer across Connecticut from 2013–2019, we found post-diagnosis weight trajectories differed by demographic and clinical characteristics. Younger age, underweight and normal BMI at diagnosis, and ER/PR−, HER2+ tumors were associated with the greatest weight gain, while older age and obesity were associated with the greatest weight loss. Large weight loss in the first year after diagnosis was strongly associated with increased risk of breast cancer-specific and all-cause mortality and moderate weight gain was modestly adversely associated with mortality. Underweight and obesity at diagnosis were also associated with a higher risk of all-cause mortality compared with normal BMI at diagnosis.

The weight gain over 5 years among women ≤45 years in our cohort (+1.75 kg) is consistent with two smaller studies^6,28^. One study of 271 breast cancer patients with 5 years of follow-up found premenopausal women (defined as <50 years) gained 1.3 kg^28^. Relatedly, a study of 625 breast cancer patients found premenopausal status was significantly associated with >2 kg weight gain during the 2 years after diagnosis^6^. Though chemotherapy-associated amenorrhea is considered a main driver of weight gain among premenopausal women, the weight gain observed in our cohort did not appear to be modified by chemotherapy receipt^33,34^. Increasingly, young women with breast cancer are being treated with therapies other than chemotherapy that can induce menopause by suppressing ovarian function, thus future research efforts should focus on understanding weight gain among this population^35^.

We also observed greater weight gain among women with ER/PR−, HER2+ tumors compared with ER/PR+, HER2−. There is limited evidence on body weight change among women with ER/PR−, HER2+ tumors; however, a recent secondary analysis of women enrolled in the ALTTO BIG 2–06 Trial evaluating chemotherapy plus trastuzumab and/or lapatinib, found 28% of women gained >5% body weight 2 years after starting anti-HER2 targeted therapy^36^. It is unclear why women with ER/PR−, HER2+ tumors gained the most post-diagnosis weight in our population, but this finding should be interpreted with caution given the small sample size of HER2+ tumors (3.5% of cases). Future research should explore post-diagnosis weight change among larger cohorts of women with ER/PR−, HER2+ breast cancer. Consistent with prior studies, we found women who had an underweight or normal BMI at diagnosis had the greatest weight gain post-diagnosis^37,38^. The magnitude of weight gain we observed among certain subgroups in our cohort exceeds that of average adult females in the United States (0.54 kg/year)^39^. These findings highlight subgroups of breast cancer patients who are most at risk for large post-diagnosis weight gain and, in turn, support increasing awareness and monitoring of post-diagnosis weight change for these populations.

Among women with stages I–III disease, we found moderate weight gain (5–10%) 1-year after diagnosis was associated with an increased risk of all-cause mortality. Similarly, a meta-analysis of 12 studies found a 12% higher risk of all-cause mortality for ≥5% body weight gain (on average 1.5 years post-diagnosis) compared with weight stability^14^. However, their additional analyses showed this association was only apparent with weight gain ≥10% (HR = 1.23, 95% CI = 1.09–1.39)^14^. Only 4.5% of our cohort gained ≥10%, thus, our power may have been limited to detect an association between large weight gain and mortality. Comparatively, a recent study of 12,590 women diagnosed with stages I–III breast cancer at Kaiser Permanente found no meaningful relationship between post-diagnosis weight gain of 5–10% (HR = 0.96, 95% CI = 0.78–1.19) or ≥10% (HR = 0.98, 95% CI = 0.74–1.31) and all-cause mortality^24^. While findings examining the relationship between weight gain and mortality among women with breast cancer are inconsistent, weight gain can negatively impact the quality of life, cause psychological distress through body image changes, and increase the risk of comorbidities^40,41^. Thus, identifying effective weight management strategies to prevent post-diagnosis weight gain is critical and has the potential to improve long-term health outcomes.

We were unable to differentiate between intentional and unintentional weight loss, as well as body composition changes (i.e., changes in muscle vs. adiposity) in our sample and acknowledge the potentially different prognostic effects of these weight loss patterns. Thus, we propose a few possible explanations for the observed weight loss across subgroups. The weight loss among older women in our cohort may be partially attributable to sarcopenia, the depletion of skeletal muscle^42^. Compounding age-related muscle atrophy, studies have shown that cancer and treatment can exacerbate the loss of lean body mass^43,44^. Sarcopenia may affect up to 45% of women with breast cancer and is associated with worse physical function, chemotoxicity and worse survival^17,42,45^. Cancer treatment can also cause nausea, vomiting, oral mucositis, dysphagia, and decreased appetite making it difficult to eat a healthy diet, which can lead to malnutrition^46^. Several exercise and nutrition interventions have improved lean body mass and reversed sarcopenia among women with breast cancer^45,47–49^. Thus, earlier identification of sarcopenia is needed as well as interventions that focus on preventing weight loss through managing nutrition impact symptoms and promoting physical activity to preserve muscle mass.

Approximately 40% of our cohort was classified as having obesity at diagnosis and a BMI ≥ 35 kg/m^2^ was associated with a 28% higher risk of all-cause mortality compared with a BMI between 18.5–25 kg/m^2^. Similar to prior studies, we found this group experienced the greatest weight loss after diagnosis (average 2–4.5 kg over 5 years)^6,20,38^. Breast cancer patients with obesity experience higher rates of surgical complications such as lymphedema, wound infections, poor wound healing and higher levels of fatigue and neuropathy, all of which may influence weight loss^50–52^. Also, sarcopenic obesity (i.e., depletion of muscle mass in combination with gains in body fat) may contribute to the weight loss observed^53^. This condition may affect 12–40% of breast cancer patients and is associated with poorer functional status and lower survival rates^54,55^. Further discerning how body composition changes during treatment among women with obesity will be critical for designing safe and effective weight management interventions in oncology care.

Many observational studies and lifestyle trials have shown diet and physical activity induced weight loss among breast cancer survivors in the post-treatment setting is feasible and effective for important health outcomes^56–59^. As a result, breast cancer survivorship guidelines promote maintaining a body healthy weight during cancer survivorship^60,61^. Our results indicate that weight changes during the first year after diagnosis, which includes active treatment, may negatively impact survival outcomes; though the intentionality of weight change was not measured. An observational study by Cespedes Feliciano et al. reported modest weight loss (>5% to <10%) within 3 years after diagnosis was associated with worse survival while modest weight loss after 3 years post-diagnosis was not associated with poorer survival, indicating early weight loss after diagnosis may be important to monitor for breast cancer outcomes^24^. However, few trials have been conducted on the prevention of weight gain or intentional weight loss during chemotherapy^62^, and the most recent American Society of Clinical Oncology report described the evidence for interventions on intentional weight loss or prevention of weight gain during active treatment as insufficient^63^. Taken together, it could be important for future research efforts to focus on designing and testing interventions for preventing weight gain and promoting healthy weight loss earlier after diagnosis and potentially during active treatment.

Our study has several noteworthy strengths, including being one of the largest to examine weight trends after breast cancer diagnosis using longitudinal methods. Our diverse sample of all women diagnosed with stages I–III breast cancer from 2013–2019 within the Smilow Cancer Network spanning Connecticut makes our results generalizable to the catchment area of our comprehensive cancer center. Using longitudinal analysis methods allowed us to detect nonlinear weight patterns. Lastly, clinically measured weight data reduced the social desirability bias of self-reported weight.

Research utilizing EHR data has inherent limitations due to how and which data are collected. For this analysis, we had more limited covariates than studies with self-reported questionnaires (e.g., exercise, diet, education, income, experiences of racism and discrimination, and other structural and society factors). Furthermore, we were unable to determine whether race and ethnicity recorded in the YTR were provided by the individual or inferred by the medical staff, thus, misclassification is likely. We also acknowledge these data are capturing social rather than biological constructs^64^. However, given limited data in the EHR, we chose to include race and ethnicity in our models as markers of risk for other factors (e.g., racism) that could be related to weight changes after breast cancer and relationships between BMI and weight change with survival. Additional research is needed to examine associations between racism, weight and cancer outcomes^65,66^. The results of our survival analyses should be considered exploratory given the small sample sizes. We had limited power to evaluate 5-year percent weight change in relation to mortality. Finally, despite our efforts to reduce potential confounding by unmeasured disease severity in our survival analyses by restricting to patients who survived at least 1 year after diagnosis and excluding women with advanced stage, this bias could still be present.

Our results update the existing literature by characterizing weight patterns after a breast cancer diagnosis under more current treatment regimens and highlight subgroups at risk for weight change over 5 years post-diagnosis. Overall, women with breast cancer from a large healthcare system experienced modest weight changes after diagnosis, though weight trajectories differed by demographic and clinical characteristics. Monitoring weight changes in the year after diagnosis and early intervention for weight management should be incorporated into clinical care. Better characterization of the changes in muscle and adiposity following a cancer diagnosis may inform strategies for tailoring weight management and lifestyle counseling services to women with breast cancer.

## Methods

### Study population

We retrospectively collected individual-level patient data from EHRs at the Smilow Care Network in Connecticut, including Smilow Cancer Hospital and 14 regional Care Centers. Breast cancer cases were ascertained from the Yale Tumor Registry (YTR) and matched by medical record number to the EHR. We identified all adult (>18 years) females diagnosed with primary stages I-III breast cancer between January 1, 2013, and June 27, 2019 (*N* = 5441). The study complied with all ethical regulations including the Declaration of Helsinki and was approved by the Yale University Human Investigations Committee (HIC: 2000027471). This medical record-based study did not require written informed consent.

### Anthropometric measures

All available body weight and height measures were extracted from the EHR for all clinic visits following each woman’s diagnosis of breast cancer during our follow-up period (January 1, 2013, to April 26, 2020). Duplicate and biological implausible weight measurements were flagged and removed using the growthcleanr package (https://carriedaymont.github.io/growthcleanr/index.html) and doublechecked by hand by two authors (L.S.P. and B.C.)^67^. Due to the variability of height in the EHR, we used the most frequently recorded height (mode).

We calculated BMI at diagnosis and categorized this according to the Centers for Disease Control and Prevention definition: underweight (≤18.5 kg/m^2^), normal weight (18.5–25.0 kg/m^2^), overweight (>25.0–30.0 kg/m^2^), Class I obesity (>30.0–35.0 kg/m^2^) or Class II/III obesity (≥35 kg/m^2^)^68^.

### Mortality data

We obtained information on deaths related to breast cancer (*N* = 209) and from all causes (*N* = 632) through March 7, 2023, from the Connecticut Tumor Registry and YTR, which captures data from Connecticut State Death Certificates, the Social Security Death Index, and the EHR.

### Covariates

Clinical and demographic characteristics, including race and ethnicity, age at diagnosis, date of diagnosis, tumor stage, estrogen receptor status, progesterone receptor status, human epidermal growth factor receptor 2 (HER2) status, surgery, chemotherapy, radiation, and endocrine therapy were abstracted from the YTR. The race and ethnicity categories (Non-Hispanic white; Non-Hispanic Black; Hispanic and Other/Unknown) were those used by the YTR. These race and ethnicity data were included to serve as markers of risk for other factors that were not available in the EHR, but could be associated with these social constructs and may be related to weight change and survival. Dates of clinical visits and pregnancy status were obtained from the EHR.

### Statistical analysis

The following exclusions were made in a stepwise fashion: (1) pregnancy noted in the EHR in the 5-year period after diagnosis, (2) every clinic visit flagged as implausible by the growthcleanr package, (3) missing height data, (4) only 1 clinic visit in the EHR post-diagnosis, (5) greater than 6 months between date of diagnosis and first clinic visit and (6) women diagnosed with stage 0 and stage IV disease. Of the 7349 women identified, 5441 were included in the analyses.

To utilize all repeated body weight measurements, we described weight trajectories 5 years post-diagnosis by fitting multiple mixed-effects regression models and obtaining estimates of weight change at 1 and 5 years post-diagnosis. Body weight at diagnosis was defined as the first clinically measured body weight within 6 months of the incident breast cancer diagnosis. Follow-up time was defined as the date of the first clinic visit to the last clinic visit. To assess the potential for nonlinearity, we compared nested models using the likelihood ratio test (models with a linear time term and linear and quadratic time terms). The final overall weight change model included both linear and quadratic time terms and a random intercept to account for within-subject correlation among repeated measures.

To explore baseline characteristics as moderators of the weight trajectories, we included a random intercept, time as linear and quadratic terms, and interaction between time and the moderating variable. The time by moderator interaction term was included in the model to account for differences in the slopes of weight change over time by moderating variables and the quadratic time by moderator interaction term accounted for differences in the shapes of the curves across time. Time-invariant covariates were included in the models as fixed effects. To account for potential confounding by unmeasured disease severity and differing treatment protocols, we included only women with stages I–III disease but performed a sensitivity analysis including stage 0 and stage IV disease.

To explore the impact of weight change on mortality, we created a five-level categorization of the percentage of weight change at 1 year post-diagnosis: weight stable (±5% of diagnosis body weight-reference group), moderate weight loss (5% to <10% loss from diagnosis body weight), large weight loss (≥10% loss from diagnosis body weight), moderate weight gain (5% to <10% gain from diagnosis body weight), and large weight gain (≥10% gain from diagnosis body weight). These weight change categories have been associated with clinically meaningful changes for reducing the risk of obesity, heart disease, diabetes and cancer^69–71^. Weight change at 1 year was defined as the measured weight closest to 1 year (within 3 months). Percent weight change was calculated as:

1 $$\frac{{Weight}\,{at}\,{one}\,{year}-{Weight}\,{at}\,{diagnosis}}{{Weight}\,{at}\,{diagnosis}}* 100$$

Only women who survived one year were included in this analysis. We used Cox proportional hazards regression models and estimated adjusted hazard ratios (HRs) and 95% confidence intervals (CIs) for each weight change category. Follow-up time for the survival analyses was the time from diagnosis to the date of death or end of the study period (March 7, 2023). For breast cancer-specific mortality, we used a cause-specific hazard function to model breast cancer-specific death simultaneously with the competing risk of death from other causes. Women who died of other causes were coded as a separate censoring event rather than true censoring^72^. The proportional hazards assumption was violated, so we additionally report the association partitioned by median follow-up time for the full sample. We explored effect modification with interaction terms of weight change category and each covariate for the all-cause mortality model, and there was no evidence of interaction. We were unable to explore effect modification in the breast cancer-specific mortality model due to the small number of individuals within each weight change strata. A sensitivity analysis was performed which included women with stage 0 and stage IV disease. We also ran a restricted cubic spline model to explore a nonlinear relationship between percent weight change at 1-year and mortality^73^. Nonlinearity was evaluated with the likelihood ratio test comparing the model with only the linear term to the model with the linear and the cubic spline terms.

We examined BMI at diagnosis in relation to all-cause and breast cancer-specific mortality using Cox proportional hazards regression models with the same follow-up time described above. We explored effect modification with interaction terms of the BMI group and each covariate for the overall survival model, and there was no evidence of interaction. The proportional hazards assumption was not violated for these models. Sensitivity analyses were performed which included women who died in the first year after diagnosis and women with stage 0 and IV disease.

Analyses were conducted using SAS Software Version 9.4 (SAS Institute Inc., Cary NC) and R version 9.0. Statistical significance was set at *p* < 0.05, two-sided.

### Reporting summary

Further information on research design is available in the Nature Research Reporting Summary linked to this article.

### Supplementary information

Supplementary Tables

Reporting Summary

## Data Availability

The datasets used and/or analyzed during the current study can be made available from the corresponding author upon reasonable request.

## Code availability

The codes developed during this study are not publicly available but may be made available to qualified researchers upon reasonable request from the corresponding author. Analyses were performed using SAS software 9.4 (Cary, NC) and R version 9.
