# Supplementary material for: Post-diagnosis weight trajectories and mortality among women with breast cancer
Source: NPJ Breast Cancer. 2023 Dec 2;9:98. doi: 10.1038/s41523-023-00603-5 (PMC10693588; doi:10.1038/s41523-023-00603-5)
Supplement: Supplementary file 1 — Supplementary Tables [file 41523_2023_603_MOESM1_ESM.pdf]

Supplementary Table 1. Weight at breast cancer diagnosis and weight change 1-year and 5-years post-diagnosis, including stages 0 and IV

	**Weight at dx, mean kg (se)[1,2]**	***p-value***	**Weight change in kg, dx to 1-year[2]**	***p-value***	**Weight change in kg, dx to 5-years[2]**	***p-value***
**Age at Diagnosis, years**						
≤45	72.2 (0.8)	Ref.	+0.91 (0.04)	Ref.	+1.76 (0.09)	Ref.
45-55	75.8 (0.6)	<0.001	+0.26 (0.03)	<0.001	+0.61 (0.06)	<0.001
55-70	77.2 (0.6)	<0.001	-0.16 (0.02)	<0.001	-1.17 (0.05)	<0.001
≥70	73.9 (0.7)	0.05	-0.88 (0.03)	<0.001	-4.20 (0.07)	<0.001
**BMI Group at Diagnosis, kg/m^2**						
≤18.5	44.6 (1.1)	<0.001	+0.66 (0.13)	0.53	+2.91 (0.30)	<0.001
18.5-25	57.3 (0.3)	Ref.	+0.57 (0.03)	Ref.	+1.75 (0.06)	Ref.
25-30	69.5 (0.3)	<0.001	+0.29 (0.03)	<0.001	-0.15 (0.06)	<0.001
30-35	82.0 (0.4)	<0.001	-0.40 (0.03)	<0.001	-2.36 (0.07)	<0.001
≥35	101.5 (0.4)	<0.001	-1.06 (0.03)	<0.001	-4.46 (0.07)	<0.001
**Race and Ethnicity**						
Non-Hispanic White	74.4 (0.4)	Ref.	+0.04 (0.02)	Ref.	-0.75 (0.04)	Ref.
Non-Hispanic Black	85.0 (0.8)	<0.001	-0.49 (0.04)	<0.001	-2.08 (0.09)	<0.001
Hispanic	74.6 (0.9)	0.83	-0.12 (0.05)	0.004	-0.85 (0.11)	0.45
Other/Unknown	67.5 (1.1)	<0.001	-0.34 (0.08)	<0.001	-0.64 (0.16)	0.51
**Clinical Stage at Diagnosis**						
Stage 0	76.0 (0.8)	0.15	0.00 (0.04)	0.53	-0.79 (0.08)	0.61
Stage I	74.9 (0.5)	Ref.	-0.03 (0.02)	Ref.	-0.84 (0.05)	Ref.
Stage II	74.8 (0.6)	0.89	+0.02 (0.03)	0.25	-0.65 (0.06)	0.02
Stage III	75.4 (1.1)	0.62	-0.14 (0.06)	0.06	-0.03 (0.13)	<0.001
Stage IV	76.6 (1.2)	0.15	-0.77 (0.07)	<0.001	-3.32 (0.15)	<0.001
Unknown	74.3 (1.1)	0.60	+0.25 (0.09)	0.003	-4.10 (0.21)	<0.001
**Tumor Subtype**						
ER/PR+, HER2-	75.4 (0.6)	Ref.	+0.04 (0.02)	Ref.	-0.98 (0.04)	Ref.

ER/PR+, HER2+	72.7 (1.0)	0.004	+0.03 (0.05)	0.91	+0.13 (0.11)	<0.001
ER/PR-, HER2+	75.6 (1.3)	0.86	+0.59 (0.07)	<0.001	+3.54 (0.45)	<0.001
TNBC	76.5 (0.9)	0.28	-0.70 (0.05)	<0.001	-1.75 (0.11)	<0.001
Unknown	75.7 (0.7)	0.61	-0.20 (0.03)	<0.001	-1.05 (0.08)	0.42
**Chemotherapy**						
No	75.1 (0.6)	Ref.	-0.12 (0.02)	Ref.	-1.65 (0.04)	Ref.
Yes	75.4 (0.6)	0.51	+0.01 (0.02)	<0.001	0.00 (0.05)	<0.001
**Radiation**						
No	73.7 (0.6)	Ref.	-0.10 (0.02)	Ref.	-1.04 (0.06)	Ref.
Yes	76.8 (0.6)	<0.001	-0.02 (0.02)	0.01	-0.84 (0.04)	0.003

Abbreviations: Dx: Diagnosis, BMI: Body mass index, TNBC: Triple-negative breast cancer.

[1]Weight at dx is based on having a first weight within 6-months of dx.

[2]Adjusted for Age at diagnosis (continuous), Race/ethnicity, Tumor subtype (ER/PR+, HER2-; ER/PR+, HER2+; ER/PR-, HER2+, TNBC), Clinical stage, Chemotherapy (yes/no), Radiation (yes/no), Endocrine therapy (yes/no).

Supplementary Table 2. Percent weight change from breast cancer diagnosis to 1-year postdiagnosis by selected characteristics, N=4,880

	Large Loss (≥10%)	Moderate Loss (5-10%)	Weight Stable (Within 5%)	Moderate Gain (5-10%)	Large Gain (≥10%)
	N (%)	N (%)	N (%)	N (%)	N (%)
**Overall**	355 (7.3)	631 (12.9)	3079 (63.1)	590 (12.1)	225 (4.5)
**Age, years**					
≤45	31 (5.9)	50 (9.5)	290 (55.3)	97 (18.5)	56 (10.7)
45-55	88 (7.1)	167 (13.5)	732 (59.0)	183 (14.8)	71 (5.7)
55-70	148 (7.6)	245 (12.6)	1271 (65.4)	206 (10.6)	75 (3.9)
≥70	88 (7.5)	169 (14.4)	786 (67.2)	104 (8.9)	23 (2.0)
**BMI, kg/m^2**					
≤18.5	1 (1.6)	2 (3.2)	41 (66.1)	13 (21.0)	5 (8.1)
18.5-25	57 (4.1)	156 (11.2)	875 (63.0)	208 (15.0)	92 (6.6)
25-30	100 (6.7)	183 (12.2)	939 (62.8)	205 (13.7)	68 (4.6)
30-35	89 (8.7)	139 (13.6)	668 (65.2)	88 (8.6)	41 (4.0)
≥35	108 (11.9)	151 (16.6)	556 (61.1)	76 (8.4)	19 (2.1)
**Race and Ethnicity**					
Non-Hispanic white	278 (7.1)	475 (12.1)	2505 (64.0)	481 (12.3)	173 (4.4)
Non-Hispanic Black	48 (11.0)	75 (17.2)	236 (54.3)	56 (12.9)	20 (4.6)
Hispanic	18 (5.5)	52 (15.8)	204 (62.0)	35 (10.6)	20 (6.1)
Other/Unknown	11 (5.4)	29 (14.2)	134 (65.7)	18 (8.8)	12 (5.9)
**Clinical Stage**					
Stage I	163 (5.2)	354 (11.4)	2076 (66.7)	393 (12.6)	128 (4.1)
Stage II	132 (10.8)	183 (15.0)	683 (56.1)	151 (12.4)	69 (5.7)
Stage III	53 (18.3)	58 (20.0)	140 (48.3)	22 (7.6)	17 (5.9)
Unknown	7 (2.7)	36 (14.0)	180 (69.8)	24 (9.3)	11 (4.3)
**Tumor Subtype**					
ER/PR+, HER2-	206 (6.1)	391 (11.6)	2209 (65.6)	422 (12.5)	142 (4.2)
ER/PR+, HER2+	30 (8.0)	54 (14.3)	223 (59.2)	48 (12.7)	22 (5.8)

ER/PR-, HER2+	29 (15.9)	36 (19.8)	87 (47.8)	19 (10.4)	11 (6.0)
TNBC	58 (12.2)	72 (15.2)	264 (55.7)	52 (11.0)	28 (5.9)
Unknown	32 (6.7)	78 (16.4)	296 (62.1)	49 (10.3)	22 (4.6)
**Chemo**					
No	130 (4.5)	317 (11.1)	1978 (68.9)	345 (12.0)	99 (3.5)
Yes	225 (11.2)	314 (15.6)	1101 (54.8)	245 (12.2)	126 (6.3)
**Radiation**					
No	130 (7.6)	250 (14.6)	1043 (60.9)	198 (11.6)	91 (5.3)
Yes	225 (7.1)	381 (12.0)	2036 (64.3)	392 (12.4)	134 (4.2)

Supplementary Table 3. Cox proportional hazard model for weight change from diagnosis to 1-year with breast cancer specific and all-cause mortality, including stage 0 and IV, N=6,117

	**Large Loss (≥10%)**	**Moderate Loss (5%-10%)**	**Stable (within 5%)**	**Moderate Gain (5%-10%)**	**Large Gain (≥10%)**
**Breast cancer-specific**	**Events/N**	**Events/N**	**Events/N**	**Events/N**	**Events/N**
251/6,117	54/430	38/739	112/3,927	30/730	17/291
Unadjusted	5.20 (3.76-7.19)	1.87 (1.29-2.70)	Ref.	1.44 (0.96-2.15)	2.08 (1.25-3.47)
Adjusted[1]	2.27 (1.62-3.20)	1.22 (0.84-1.80)	Ref.	1.34 (0.89-2.00)	1.09 (0.65-1.80)
**All-cause mortality**	**Events/N**	**Events/N**	**Events/N**	**Events/N**	**Events/N**
684/6,117	131/430	100/739	346/3,927	72/730	35/291
Unadjusted	4.28 (3.50-5.23)	1.63 (1.31-2.04)	Ref.	1.11 (0.86-1.44)	1.39 (0.98-1.97)
Adjusted[1]	2.75 (2.22-3.40)	1.29 (1.03-1.62)	Ref.	1.31 (1.01-1.70)	1.15 (0.81-1.64)

[1]Adjusted for Age at diagnosis (continuous), BMI at diagnosis (continuous), Race and Ethnicity, Tumor subtype (ER/PR+, HER2-; ER/PR+, HER2+; ER/PR-, HER2+, TNBC), Clinical stage, Chemotherapy (yes/no), and Radiation (yes/no).

Supplementary Table 4. Cox proportional hazard model for weight change from diagnosis to 1-year and all-cause mortality partitioned by follow-up time, including stages 0 and IV

	All-cause mortality	Adjusted[1] HR (95% CI)	p-value
		**<6 Years of Follow-up**	
**% Weight change**	589/2566		
Large Loss vs. Stable	121/252	2.08 (1.66-2.61)	<0.001
Moderate Loss vs. Stable	83/349	1.05 (0.82-1.34)	0.71
Moderate Gain vs. Stable	61/287	1.13 (0.85-1.49)	0.40
Large Gain vs. Stable	29/115	1.02 (0.69-1.50)	0.94
		**≥ 6 Years of Follow-up**	
**% Weight change**	95/3551		
Large Loss vs. Stable	10/178	3.23 (1.56-6.7)	0.002
Moderate Loss vs. Stable	17/390	2.12 (1.21-3.7)	0.008
Moderate Gain vs. Stable	11/443	1.89 (0.97-3.7)	0.06
Large Gain vs. Stable	6/176	2.54 (1.05-6.1)	0.04

[1]Adjusted for Age at diagnosis (continuous), BMI at diagnosis (continuous), Race and Ethnicity, Tumor subtype (ER/PR+, HER2-; ER/PR+, HER2+; ER/PR-, HER2+, TNBC), Clinical stage, Chemotherapy (yes/no), and Radiation (yes/no).

Supplementary Table 5. Association between BMI at diagnosis and breast cancer-specific and all-cause mortality via a Cox proportional hazards model, including stages 0 and IV and women who died in the first year after follow-up, N=6,934

	**All-cause mortality (854/6,934)**	**Adjusted[1] HR (95% CI)**	***p-value***	**Breast cancer-specific mortality (333/6,934)**	**Adjusted[1] HR (95% CI)**	***p-value***
**BMI at Diagnosis, kg/m²**						
≤18.5 vs. 18.5-25	18/85	2.53 (1.56-4.10)	<0.001	9/85	2.96 (1.48-5.92)	0.002
25-30 vs. 18.5-25	235/2120	0.94 (0.78-1.14)	0.54	101/2120	1.10 (0.81-1.48)	0.55
30-35 vs. 18.5-25	193/1446	1.04 (0.86-1.27)	0.67	76/1446	1.10 (0.80-1.51)	0.57
≥35 vs. 18.5-25	193/1279	1.34 (1.10-1.63)	0.004	69/1279	1.18 (0.85-1.64)	0.32
**Continuous, per 5 kg/m²**		1.06 (1.01-1.11)	0.03		0.98 (0.90-1.06)	0.62

[1]Adjusted for Age at diagnosis (continuous), Race and Ethnicity, Tumor subtype (ER/PR+, HER2-; ER/PR+, HER2+; ER/PR-, HER2+, TNBC), Clinical stage, Chemotherapy (yes/no), and Radiation (yes/no).

Supplementary Table 6. Association between BMI at diagnosis and all-cause mortality via a Cox proportional hazards model, including stage 0 and IV, excluding women who died in the first year after diagnosis, N=6,821

	**All-Cause Mortality (N=741/6,821)**	**Adjusted[1] HR (95% CI)**	***p-value***	**Breast cancer-specific mortality (N=271/6,821)**	**Adjusted[1] HR (95% CI)**	***p-value***
**BMI, kg/m²**						
≤18.5 vs. 18.5-25	16/83	2.78 (1.66-4.65)	<0.001	7/83	3.29 (1.50-7.22)	0.003
25-30 vs. 18.5-25	196/2081	0.90 (0.73-1.10)	0.30	79/2081	1.09 (0.78-1.53)	0.61
30-35 vs. 18.5-25	173/1426	1.07 (0.87-1.32)	0.53	63/1426	1.16 (0.81-1.65)	0.42
≥35 vs. 18.5-25	168/1254	1.33 (1.08-1.65)	0.01	59/1254	1.26 (0.88-1.81)	0.20
**Continuous, per 5 kg/m²**		1.05 (0.99-1.11)	0.06		0.99 (0.91-1.08)	0.83

[1]Adjusted for Age at diagnosis (continuous), Race and Ethnicity, Tumor subtype (ER/PR+, HER2-; ER/PR+, HER2+; ER/PR-, HER2+, Triple negative), Clinical stage, Chemotherapy (yes/no), and Radiation (yes/no).
