# Supplementary material for: Post-diagnosis weight trajectories and mortality among women with breast cancer
Source: NPJ Breast Cancer. 2023 Dec 2;9:98. doi: 10.1038/s41523-023-00603-5 (PMC10693588; doi:10.1038/s41523-023-00603-5)
Supplement: Supplementary file 2 — Reporting Summary [file 41523_2023_603_MOESM2_ESM.pdf]

nature portfolio

Corresponding author(s): Leah Puklin

Last updated by author(s): Oct 31, 2023

Reporting Summary

Nature Portfolio wishes to improve the reproducibility of the work that we publish. This form provides structure for consistency and transparency in reporting. For further information on Nature Portfolio policies, see our Editorial Policies and the Editorial Policy Checklist.

Statistics

For all statistical analyses, confirm that the following items are present in the figure legend, table legend, main text, or Methods section.

n/a	Confirmed	
☐	☒	The exact sample size (*n*) for each experimental group/condition, given as a discrete number and unit of measurement
☐	☒	A statement on whether measurements were taken from distinct samples or whether the same sample was measured repeatedly
☐	☒	The statistical test(s) used AND whether they are one- or two-sided<br>*Only common tests should be described solely by name; describe more complex techniques in the Methods section.*
☐	☒	A description of all covariates tested
☐	☒	A description of any assumptions or corrections, such as tests of normality and adjustment for multiple comparisons
☐	☒	A full description of the statistical parameters including central tendency (e.g. means) or other basic estimates (e.g. regression coefficient) AND variation (e.g. standard deviation) or associated estimates of uncertainty (e.g. confidence intervals)
☐	☒	For null hypothesis testing, the test statistic (e.g. F, t, r) with confidence intervals, effect sizes, degrees of freedom and P value noted<br>*Give P values as exact values whenever suitable.*
☒	☐	For Bayesian analysis, information on the choice of priors and Markov chain Monte Carlo settings
☐	☒	For hierarchical and complex designs, identification of the appropriate level for tests and full reporting of outcomes
☐	☒	Estimates of effect sizes (e.g. Cohen's *d*, Pearson's *r*), indicating how they were calculated

Our web collection on statistics for biologists contains articles on many of the points above.

Software and code

Policy information about availability of computer code

Data collection	No software was used
Data analysis	Analyses were conducted using SAS Software Version 9.4 (SAS Institute Inc., Cary NC) and R version 9.0.

For manuscripts utilizing custom algorithms or software that are central to the research but not yet described in published literature, software must be made available to editors and reviewers. We strongly encourage code deposition in a community repository (e.g. GitHub). See the Nature Portfolio guidelines for submitting code & software for further information.

Data

Policy information about availability of data

All manuscripts must include a data availability statement. This statement should provide the following information, where applicable:

- Accession codes, unique identifiers, or web links for publicly available datasets
- A description of any restrictions on data availability
- For clinical datasets or third party data, please ensure that the statement adheres to our policy

The datasets used and/or analyzed during the current study can be made available from the corresponding author upon reasonable request.

Research involving human participants, their data, or biological material

Policy information about studies with human participants or human data. See also policy information about sex, gender (identity/presentation), and sexual orientation and race, ethnicity and racism.

Reporting on sex and gender	All female breast cancer stages I-III cases diagnosed between 2013 and 2019 from Smilow Cancer Hospital, Yale New Haven Hospital
Reporting on race, ethnicity, or other socially relevant groupings	We used the race and ethnicity variable pulled directly from the Electronic Health Records and the Yale Tumor Registry. This variable is categorized as Non-Hispanic white, Non-Hispanic Black, Hispanic, Other/Unknown.
Population characteristics	We included additional covariates in our models which were also pulled directly from the electronic health records and the Yale Tumor Registry. These included age, clinical stage at diagnosis, tumor subtype, chemotherapy receipt, radiation receipt, endocrine therapy receipt.
Recruitment	We retrospectively collected individual-level patient data from the Electronic Health Records (EHRs) at the Smilow Care Network in Connecticut, including Smilow Cancer Hospital and 14 regional Care Centers. Breast cancer cases were ascertained from the Yale Tumor Registry (YTR) and matched by medical record number to the EHR. We identified all adult (>18 years) females diagnosed with a primary stage I-III breast cancer between January 1, 2013 and June 27, 2019 (N= 5,441).
Ethics oversight	The study was approved by the Yale University Human Investigations Committee. Study was medical record based so there was no written informed consent.

Note that full information on the approval of the study protocol must also be provided in the manuscript.

Field-specific reporting

Please select the one below that is the best fit for your research. If you are not sure, read the appropriate sections before making your selection.

☐ Life sciences ☒ Behavioural & social sciences ☐ Ecological, evolutionary & environmental sciences

For a reference copy of the document with all sections, see nature.com/documents/nr-reporting-summary-flat.pdf

Behavioural & social sciences study design

All studies must disclose on these points even when the disclosure is negative.

Study description	We provide a contemporary quantitative evaluation of weight trajectories over 5-years post breast cancer diagnosis in a large sample of patients from a major healthcare system in Connecticut using robust longitudinal methods and clinically measured repeated weight data from electronic health records (EHRs).
Research sample	We retrospectively collected individual-level patient data from EHRs at the Smilow Care Network in Connecticut, including Smilow Cancer Hospital and 14 regional Care Centers. Breast cancer cases were ascertained from the Yale Tumor Registry (YTR) and matched by medical record number to the EHR. We identified all adult (>18 years) females diagnosed with a primary stages I-III breast cancer between January 1, 2013 and June 27, 2019 (N= 5,441).<br><br>The following exclusions were made in a stepwise fashion: 1) pregnancy noted in the EHR in the 5-year period after diagnosis, 2) every clinic visit flagged as implausible by the growthcleanr package, 3) missing height data, 4) only 1 clinic visit in the EHR post-diagnosis, and 5) greater than 6 months between date of diagnosis and first clinic visit 6) women diagnosed with stage 0 and stage IV disease. Of the 7,349 women identified, 5,411 were included in the analyses.<br><br>Among the 5,441 women with stages I-III breast cancer, the average duration of follow-up available in our dataset was 3.2 years (range=0.01-7.2 years) with a mean of 34.4±25.5 (± standard deviation [SD]) weight measurements (Figure 1 and Table 1). At diagnosis, the mean age was 61.0±13.2 years and mean BMI was 29.3±6.9 kg/m2. Most patients were Non-Hispanic white (80.3%), had stage I disease (63.7%), had ER/PR+, HER2- tumors (68.5%), did not receive chemotherapy (60.6%), received radiation therapy (62.9%), and had breast conserving surgery (63.1%).
Sampling strategy	We identified all adult (>18 years) females diagnosed with a primary stages I-III breast cancer between January 1, 2013 and June 27, 2019 (N= 5,441).
Data collection	We retrospectively collected individual-level patient data from EHRs at the Smilow Care Network in Connecticut, including Smilow Cancer Hospital and 14 regional Care Centers.
Timing	The female diagnoses were between January 1, 2013 and June 27, 2019. Follow-up time was from January 1, 2013 to April 26, 2020
Data exclusions	The following exclusions were made in a stepwise fashion: 1) pregnancy noted in the EHR in the 5-year period after diagnosis, 2) every clinic visit flagged as implausible by the growthcleanr package, 3) missing height data, 4) only 1 clinic visit in the EHR post-diagnosis, and 5) greater than 6 months between date of diagnosis and first clinic visit 6) women diagnosed with stage 0 and stage IV disease. Of the 7,349 women identified, 5,411 were included in the analyses.

Non-participation: N/A

Randomization: No randomization

Reporting for specific materials, systems and methods

We require information from authors about some types of materials, experimental systems and methods used in many studies. Here, indicate whether each material, system or method listed is relevant to your study. If you are not sure if a list item applies to your research, read the appropriate section before selecting a response.

Materials & experimental systems

n/a	Involved in the study
☒	☐ Antibodies
☒	☐ Eukaryotic cell lines
☒	☐ Palaeontology and archaeology
☒	☐ Animals and other organisms
☒	☐ Clinical data
☒	☐ Dual use research of concern
☒	☐ Plants

Methods

n/a	Involved in the study
☒	☐ ChIP-seq
☒	☐ Flow cytometry
☒	☐ MRI-based neuroimaging
